# Genome Wide Distributions and Functional Characterization of Copy Number Variations between Chinese and Western Pigs

**DOI:** 10.1371/journal.pone.0131522

**Published:** 2015-07-08

**Authors:** Hongyang Wang, Chao Wang, Kui Yang, Jing Liu, Yu Zhang, Yanan Wang, Xuewen Xu, Jennifer J. Michal, Zhihua Jiang, Bang Liu

**Affiliations:** 1 Key Laboratory of Agricultural Animal Genetics, Breeding and Reproduction of Ministry of Education, Huazhong Agricultural University, Wuhan, PR China; 2 The Cooperative Innovation Center for Sustainable Pig Production, Huazhong Agricultural University, Wuhan, PR China; 3 Modern Educational & Technology Centre of Huazhong Agricultural University, Wuhan, PR China; 4 Department of Animal Sciences, Washington State University, Pullman, WA, United States of America; China Agricultural Univeristy, CHINA

## Abstract

Copy number variations (CNVs) refer to large insertions, deletions and duplications in the genomic structure ranging from one thousand to several million bases in size. Since the development of next generation sequencing technology, several methods have been well built for detection of copy number variations with high credibility and accuracy. Evidence has shown that CNV occurring in gene region could lead to phenotypic changes due to the alteration in gene structure and dosage. However, it still remains unexplored whether CNVs underlie the phenotypic differences between Chinese and Western domestic pigs. Based on the read-depth methods, we investigated copy number variations using 49 individuals derived from both Chinese and Western pig breeds. A total of 3,131 copy number variation regions (CNVRs) were identified with an average size of 13.4 Kb in all individuals during domestication, harboring 1,363 genes. Among them, 129 and 147 CNVRs were Chinese and Western pig specific, respectively. Gene functional enrichments revealed that these CNVRs contribute to strong disease resistance and high prolificacy in Chinese domestic pigs, but strong muscle tissue development in Western domestic pigs. This finding is strongly consistent with the morphologic characteristics of Chinese and Western pigs, indicating that these group-specific CNVRs might have been preserved by artificial selection for the favored phenotypes during independent domestication of Chinese and Western pigs. In this study, we built high-resolution CNV maps in several domestic pig breeds and discovered the group specific CNVs by comparing Chinese and Western pigs, which could provide new insight into genomic variations during pigs’ independent domestication, and facilitate further functional studies of CNV-associated genes.

## Introduction

Genomic structural variations due to large insertions, deletions, inversions and translocations are called copy number variations (CNVs). The genome region CNV happened is defined CNV region (CNVR), which can range in size from one Kb to several Mb and occur with high frequency in genomes [1]. Until now, CNVs have been detected in humans [2–4], mice [5, 6], dogs [7, 8], pigs [9–12], cattle [13–15] and chickens [16, 17].

Copy number variations have been linked to many diseases, disorders, and phenotypic traits. In humans a set of CNVs was present in 189 subjects diagnosed with major depressive disorder who previously attempted suicide compared to 1,073 subjects with major depressive disorder but never attempted suicide [18], while Liu *et al*. [19] found that a deletion at 2p24.3 was significantly associated with prostate cancer risk in 498 aggressive prostate cancer cases using affymetrix SNP arrays. In addition, the effect of CNVs on body mass index and early-onset extreme obesity has been reported [20, 21]. In livestock animals, four types of duplications at the *KIT* locus were exclusively present in the dominant white allele, which caused white or white-spotted coat colours in pigs [22]. Another *KIT* gene related study performed a database of 50K SNP genotypes from 4,500 cattle revealed that colour sidedness was determined by translocation events between chromosomes 6 and 29 [23]. Recently, Xu *et al*. found that 34 CNVs on 22 chromosomes were associated with several milk production using 26,362 Holstein bulls and cows [24]. Furthermore, a 4.6 Kb duplication in intron 6 of the *STX17* gene leads to the greying with age phenotype in horses [25]. Disruption of the *CCDC108* gene by structural rearrangement causes a sperm motility defect in male chickens homozygous for rose-comb phenotype, while a CNV in intron 1 of *SOX5* causes the pea-comb phenotype in chickens [16, 26].

Single nucleotide polymorphism (SNP) chips and comparative genomic hybridization (CGH) arrays have served as useful tools to detect CNVs in the past [27–29]. Certainly, their limitations include low probe density and cross-hybridization of repetitive sequences, which can lead to a high number of false-positive results in CNV detection [15]. However, evidence has clearly shown that next generation sequencing (NGS) technology can significantly benefit discovery of CNVs [11, 15].Generally speaking, NGS strategies for CNV detection have mainly relied on read-depth (RD) and paired-end mapping (PEM) approaches. The PEM method is only applicable to paired-end reads [30]. Moreover, the PEM approach is suitable for detecting CNVs in regions of low complexity [31]. In contrast, RD methods depend on the depth of coverage in the genomic region to estimate the CNV value. RD methods can detect large insertions and CNVs in complex genomic regions [32].

In the present study, we focused on discovery of group-specific CNVs and their associated genes that affect disease resistance, reproduction and growth rate in domestic Chinese and Western pigs by using NGS data from 49 individuals from Chinese and Western breeds [33–35]. Here we report novel CNVs generated during the domestication of pigs. Some of the CNVs were specific to either Chinese or Western breeds, which might due to the independent domestication and trait selection differences between Chinese and Western pigs. In particular, the CNV-associated genes in the Chinese breeds were involved in the inflammatory response and reproduction, whereas, CNV-associated genes in the Western domestic pigs were related to muscle tissue development. The novel CNV information from this research is expected to enrich the data of porcine genome variations and facilitate further research on the differences between Chinese and Western domestic pig.

## Materials and Methods

### Data collection and sequence alignment

The whole genome sequencing data from 13 populations of *Sus scrofa* containing a total of 49 individuals were obtained as previously described [33–35], and all sequencing data were generated using the Illumina HiSeq platform. The libraries were 100 bp pair-end reads and the insert sizes ranged from 300–500 bp. The samples included one European wild boar (Netherlands), one Chinese wild boar (South China), twenty five Western domestic pigs from four commercial breeds and twenty two Chinese domestic pigs of seven breeds from South China (Table A in S1 File).

Before sequence alignment, repeat regions in the porcine genome (*Sus scrofa* build 10.2) were masked using RepeatMasker [36] (RepeatMasker-open-4-0-3, RMBlast as the search engine and repeatmasker libraries-20130422 as the library,-s option). Additionally, masked regions were extended 100 bp in both directions to avoid boundary alignment effects [15, 37]. Mapping of the reads to the masked porcine genome was performed with Bowtie2 (-x -1–2-S-D 15-R 2-N 0-L 22 –i S,1,1.15). Approximately 49% of the genome was masked and 50% of the raw reads were mapped to the unmasked portion of the genome. Subsequently, the SAM file was converted to the BAM file, sorted and indexed using Samtools [38]. Finally, the BAM files were strictly filtered using a high mapping quality value (≥42) to reduce spurious alignment (Table A in S1 File).

### Identification of pig CNVs

The CNVs in all individuals were identified using CNV-seq [39] and CNVnator [40] based on the RD method. Reads were counted using a sliding window approach and used to find CNVs. The CNV-seq was run under a robust statistical model and the CNVs were evaluated by comparing the test samples to the reference samples (wild boars) [39]. Therefore, the results from the CNV-seq analysis represented the CNVs generated from wild boars to domestic pigs during domestication. Window size was about 10 Kb in this process. The range of discovered CNVs was broadened by CNVnator based on the combination of the established mean-shift approach with additional refinements (multiple-bandwidth partitioning and GC correction) to span 300 bp along the whole genome of each individual [40]. Credible CNVRs between 1 Kb to 100 Kb identified by CNVseq and CNVnator were selected and combined for each individual. For analyzing CNV differences between Chinese and Western breeds, all the CNVRs from Chinese domestic pigs and Western domestic pigs were combined into two groups to represent their respective CNVs during domestication.

### Experiment validation

Primers were designed using the Primer 5.0 tool so the expected amplicon lengths were restricted between 100 and 300 bp and the GC percentages were between 40% and 60%. Primers were further tested for unique binding sites by Primer-BLAST [41]. Primer amplification efficiencies were determined by testing them on a standard curve of DNA over 5 logs of concentration (Table A in S2 File). qPCR were performed using 25 ng of pig genomic DNA as template in a final volume of 10 ul containing 5 ul SYBR Green Realtime PCR Master Mix (TOYOBO), 3.6 ul ddH2O and 15 ng of each primer. All reactions were amplified on CFX384 Real-Time System (BioRad) in triplicate. The CN values in the test loci were calculated as (1+e) ^(1-ddCt)^. The porcine glucagon gene (*GCG*) was included as the single copy control gene [9, 12]. To reduce batch and platform effects, plates were designed to amplify the reference gene and the same sample in each experiment.

### Gene content and Gene Ontology in CNVRs

The genes located in CNVRs were assessed using BioMart [42] according to Ensembl. The CNVRs and gene regions containing start and end sites of exons and introns were compared to find the CNV effects on amino acids or introns for each gene. The DAVID web tool [43, 44] and Blast2go [45] were used to identify genes in the porcine CNVRs that were homologous to human genes and to classify the genes in terms of molecular function, cell component, biological process, and pathway. All the data were considered to be statistically significant at P<0.05.

## Results and Discussion

### Detection of CNVRs in domestic pigs

**Fig 1:**
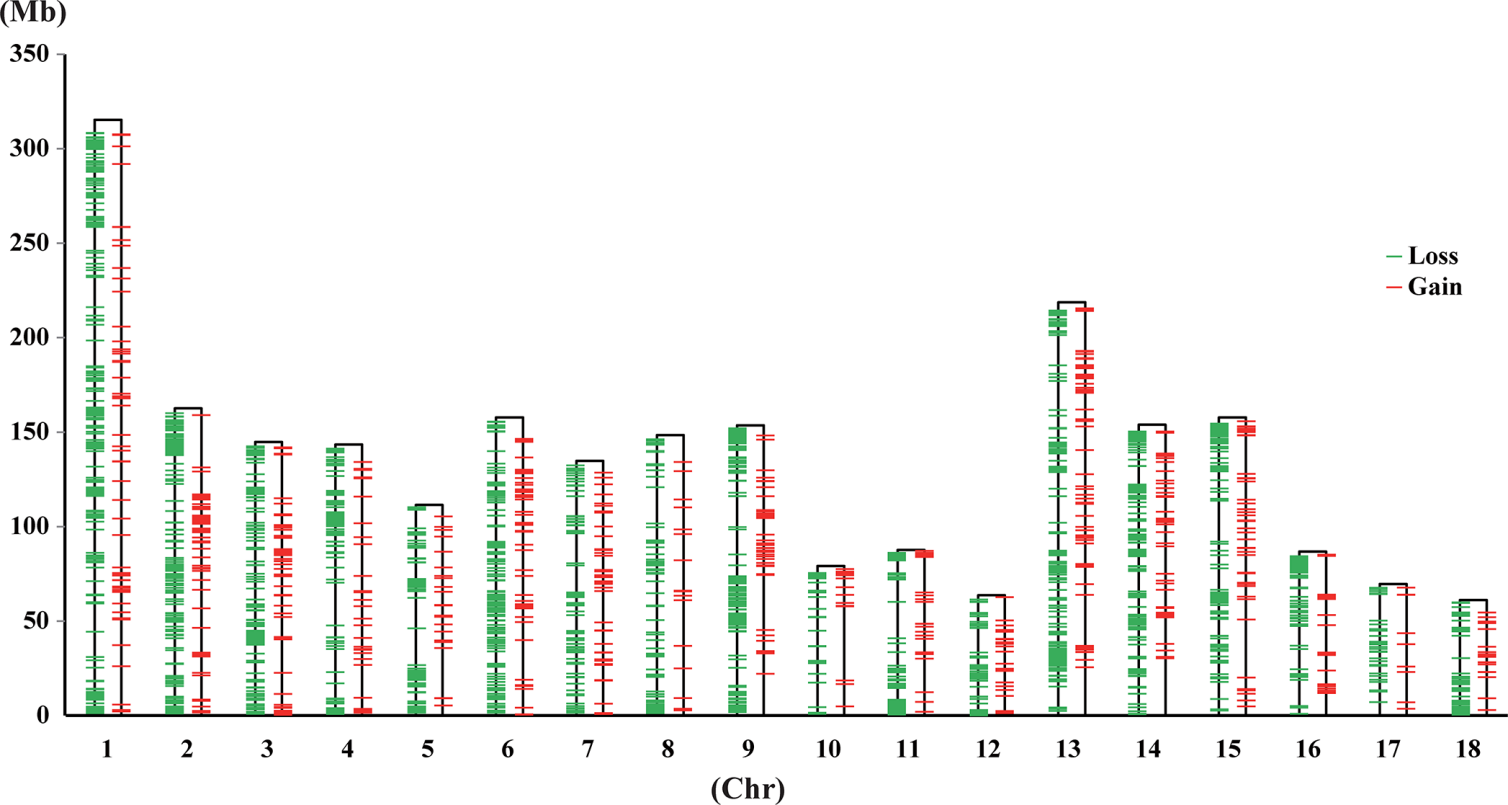
Distribution of CNVRs found in autosomes of porcine genome. X axis indicates the 18 autosomes, Y axis the length of each chromosome, and the black frames the different chromosomes. The green bars (left border of each chromosome) represent copy number loss regions and the red bars (right border of each chromosome) represent copy number gain regions.

CNV-seq [39] and CNVnator [40] were used for CNVs detection among 49 individual pigs. The combination of these two methods discovered 3,131 CNVRs based on the RD information of domestic individuals in comparison to a wild boar (one Chinese and one Western) (Table B in S1 File). The CNVRs occupied a total of 42.1 Mb or 1.72% of the pig genome (Fig 1; Table C in S1 File). The size of the CNVRs varied from 1 to 88.8 Kb, and averaged 13.4 Kb. Among the 3,131 CNVRs, 745 gained CN, 2,364 lost CN and 22 show both CN gain and loss within the same regions from different individuals as compared to the wild boar. In particular, loss variations accounted for 75% (2,364 loss/3,131 total) of CNVs, indicating that these variations may be related to the deletion of chromosome regions. The CNVR density varied from 0.75% on chromosome 17 to 2.40% on chromosome 2 (Table C in S1 File), which is consistent with a previous report by Paudel and colleagues using 16 pigs from Europe and Asia [11].

**Fig 2:**
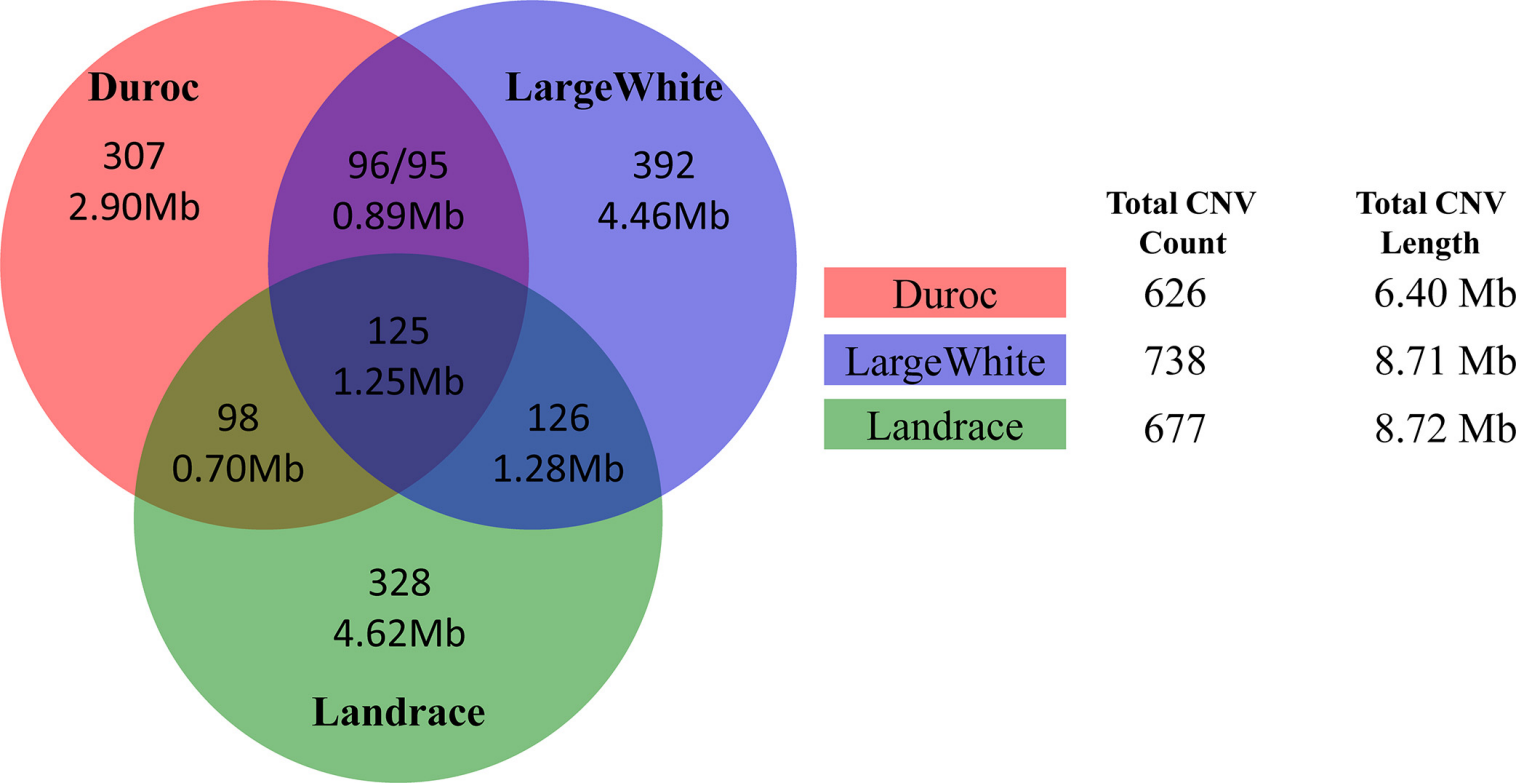
CNVs sharing intervals and basepairs among Western breeds. In the Venn diagram, the top number indicates the count of shared CNVs, and the bottom number the CNV intervals and basepairs among three Western breeds. The table to the right of the diagram shows total CNV counts and length in each breed.

We observed that the CNVR numbers varied among the 13 breeds used in the present study. Of the Chinese pig breeds, the number of CNVRs varied from 383 (12%) in Penzhou pigs to 919 (29%) in Tongcheng pigs. With respect to Western pig breeds, 738 (24%), 677 (22%), 626 (20%) and 119 (4%) CNVRs were found in LargeWhite, Landrace, Duroc and Hampshire pigs, respectively. Interestingly enough, these breeds had only a few common CNVRs (Fig 2). These results proved that there are fewer shared CNVs among different breeds, which is consistent with Bickhart *et al*. study that greatest CNV diversities are existed among five different cattle breeds [15].

After comparing our results with CNVRs previously identified from NGS data generated by other researchers, we found only a few overlapping events across the different datasets. First, we compared our CNVRs with which were identified using SNP chips, consisting of Chinese domestic pig breeds. Chen *et al*. [46] used porcine SNP60 beadchip data of 18 populations and discovered 565 non-redundant CNVRs in 1,327 individuals. Only 174 CNVRs in our results were overlapped with Chen *et al*. study. For recently study of Dong *et al*. [47], they performed PennCNV to discover CNVs with SNP60 beadchip of 96 individuals from three Chinese pig breeds. Totally 105 CNVRs were found. There’s also a few CNVRs overlapped with ours. We thought that low probe density of SNP chips might lead to a high number of false-positive results in CNV detection and most of Chinese breeds they used are Bamaxiang, Dongshan, Erhualian, Minzhu, Rongchang pigs *et al*. in Chen *et al*. study and Tibetan, Dahe and Wuzhishan pigs in Dong *et al*. study, which are different with Chinese breeds used in ours. After that, we did comparisons between our CNVRs and others used NGS data. Rubin *et al*. [22] identified 1,928 CNVRs from 8 pig breeds. Among them, only 28 overlapped our results. Paudel *et al*. used the NGS data of 16 individual pigs including European and Asian pigs and identified 3,118 CNVRs [11]. Of them, 164 regions overlapped our results. It is possible that different sample sizes and pig breeds or origins might have caused the differences in CNVRs reported by the various groups. We used a large NGS data set containing 49 individuals collected from 13 pig breeds. In comparison, Paudel used only 16 individual pigs and most were Xiang and Jiangquhai breeds of Asian pigs. Our present study did not include any Xiang and Jiangquhai pigs. In addition, we used sequence data from individual pigs, rather than pooled sequence data used by Rubin *et al*. This comparison result was consistent with the observation by Bickhart *et al*., who pointed out that the CNV differences were greater among breeds [15].

### Genomic features of CNVRs

Previous studies showed that CNVs were formed by non-allelic homologous recombination (NAHR) associated with many repetitive elements in genome [48, 49]. These repeat elements associated with the breakpoints of CNVR are often Alu and LINE retrotransposons and microsatellites [50]. As such, we merged all CNVRs and the 10 Kb flanking regions from both sides of the region and compared these sequences with the repeat element regions using the RepeatMasker software. Interestingly enough, the merged regions had more than twice the number of repeat elements (Table 1) than the genome wide average (Fisher test, P<0.001). The repeat elements included SINE (Alu, MIRs), LINE (L1, L2, etc.), LTR (ERVL-MaLR, ERV1, etc.) and other DNA elements. Based on these results, we speculate there is a high association between repeat elements and CNVRs. In particular, the repeat elements might promote the formation of CNVs [11, 51].

**Table 1:** Density and number of repeat elements in CNVRs compared to porcine genome.

Repeat element	Pig CNVRs		Porcine Genome	
	number ^a^	Density ^b^	number ^a^	Density ^c^
**LINEs**	43,642	11.321*	918,156	3.746
**SINEs**	58,738	15.237*	1,660,382	6.775
**Small RNA**	189	0.049	1,268,659	5.177
**LTR elements**	11,191	2.903*	299,047	1.220
**DNA elements**	9,713	2.520*	278,366	1.136
**Satellites**	36	0.009*	1,019	0.004
**Unclassified**	187	0.049*	5,394	0.022
**Total**	123,696	32.087*	4,431,023	18.081

^a^ Total counts of each repeat element in pig CNVRs and porcine genome.

^b^ All CNVRs and 10 Kb flanking regions in both directions of CNVRs were merged, and repeat elements were calculated at 10 Kb intervals.

^c^ Porcine genome was divided into 10 Kb regions and repeat elements were counted.

* P-value (< 0.001, χ2 tests)

### CNV validation

**Fig 3:**
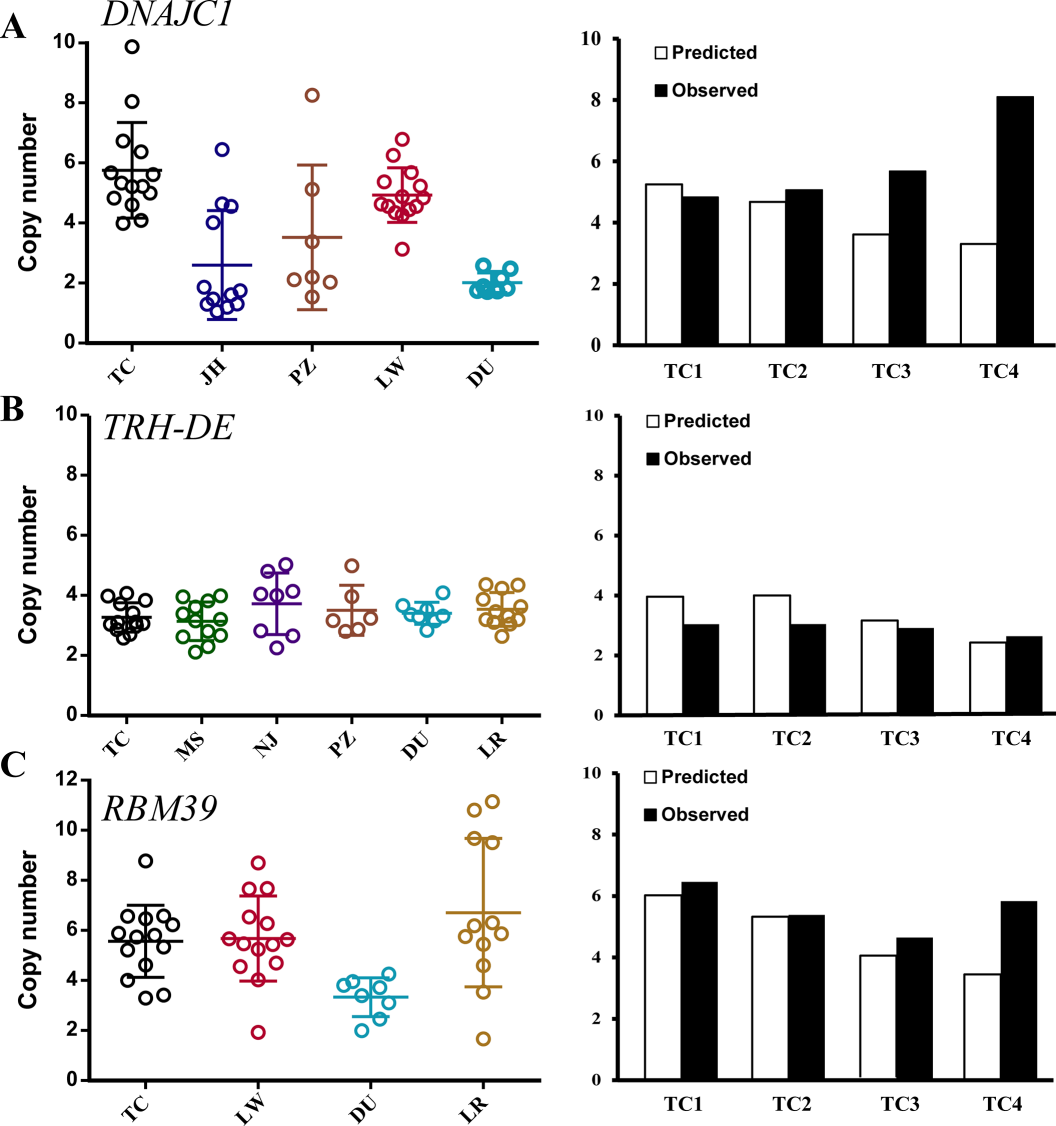
CN values predicted and observed near three gene loci. The left pictures are CN values estimated from at least 5 individuals in different breeds using the qPCR method. X axis means different pig breeds (TC = Tongcheng; MS = Meishan; NJ = Neijiang; JH = Jinhua; PZ = Penzhou; LW = LargeWhite; DU = Duroc; LR = Landrace). Y axis means the CN values. The cycles indicate CN values for each individual. The right histograms describe the predicted and observed CN values in four Tongcheng pigs. CN gain in these four gene loci was predicted. **(A) (B)** and **(C)** The left pictures showed that CN was increased in most individuals in a large range for 2–12. Also, the same trend was observed for CN gain between predicted and observed events in three Tongcheng pigs according to the right histograms.

In the present study, we selected 28 novel CNVRs including 19 genic CNVRs and 9 non-genic CNVRs for validation using quantitative real time-polymerase chain reaction (qPCR) assays. The ddCt method was used to determine the copy number of these regions in domestic pigs relative to those of wild boars. Nearly 86% (24 confirmed/28 total) of the predicted CNVRs were confirmed by qPCR (Table A in S2 File; Fig 3; S5 File), indicating a low false discovery rate of CNV calling and a high sensitivity of our qPCR method. We also validated the predicted CNVRs on chromosome 8 containing the *KIT* gene, which is associated with the dominant white color in Western pigs [22]. We plotted the log_2_ ratio CNV graph in the region using the ggplot package [39] and found a large CNV region of 43.2–43.8 Mb in LargeWhite pigs and two CNV regions of 43.3–43.4 Mb and 43.6–43.8 Mb in Hampshire pigs, respectively (S4A File). To confirm the CNVRs in LargeWhite, Landrace, and Duroc individuals, two distinct primer sets were designed for the qPCR test. Both LargeWhite and Landrace pigs showed a CN gain from 3 to 11 copies in the 43.2–43.8 Mb region and 6 to 21 copies in the 43.3–43.4 Mb region, but no CN variation appeared in Duroc individuals within these two regions (S4B File). The CNVRs found in LargeWhite and Hampshire pigs were located in the DUP1 and DUP3/4 regions, respectively, which have also been reported previously [22]. Despite the differences in the methods and materials we used, we found the same results in the *KIT* region previously reported by others [22]. Furthermore, the validation of the CNVs detected in the *KIT* gene region indicated the credibility of our CNV prediction and qPCR verification methods.

### CNV-associated genes

Among the 3,131 CNVRs described above, 1,243 CNVRs (40%) harbored a total of 1,363 genes according to the *Sus scrofa* build 10.2 assembly and Ensembl (release 76), including 1,266 protein-coding genes, 6 pseudogenes, 25 miRNA, 3 miscRNA, 4 rRNA, 18 snRNA, 12 snoRNA and 4 processed transcripts. Among the 1,243 CNVRs, 222 (18%) completely encompassed 333 genes, 484 (39%) partly overlapped with 531 genes, and 671 (54%) were located in 559 genes. Also, we found 865 CNVRs (70%) and 378 CNVRs (30%) involved in 1,079 gene exons and 320 gene introns, respectively (Table B in S2 File). Among the 1,363 genes, 899 were completely orthologous with humans according to the human orthologs of porcine genes. Gene ontology analysis indicated that CNVRs harbored genes were mainly involved in biological adhesion, GTPase regulator activity, amino acid phosphorylation, cell junction, plasma membrane part and MAPK signaling pathway (p-value < 0.05) in terms of the molecular function, cell component, biological process and pathway enrichment (Table C in S2 File).

Many genes were also found to be involved in olfaction, immunity, and lipid metabolism according to the Ensembl annotation and a previous report [52], which was consistent with the observation that olfactory and immune gene families are two large gene families associated with CNVs [11, 15] (Tables B, D and E in S2 File). Based on human orthologous genes obtained from the Online Mendelian Inheritance in Man (OMIM) database, 198 (6%) of the 3,131 CNVRs identified in the present study are associated with human orthologous OMIM genes involved in immunodeficiency, muscular dystrophy, and lipase deficiency (Table B in S1 File). These CNVRs might contribute to diseases, which need to be further validated in the future.

### Functional features of CNVR associated genes in Chinese and Western pig breeds

Diversification of artificial selection during independent domestication contributes to the characteristic differences between Asian and European pigs [33, 53]. In our study, 2,278 CNVRs (567 duplications, 1706 deletions and 5 both) and 1,547 CNVRs (315 duplications and 1223 deletions and 9 both) were found in Chinese and Western breeds, with a total CNV size of 28.9 Mb and 19.8 Mb, respectively (Tables A and B in S3 File). Among all the CNVRs, 618 were present in both Chinese and Western domestic pigs (Table C in S3 File), and the remaining CNVRs were specific to either Chinese or Western breeds. These results showed that CNVs reflected Eastern and Western origins of the domestic pigs. Considering CNV inconsistency among different breeds, we selected those shared by at least four Chinese breeds and two Western breeds (≥ 50% of total breeds), and constructed two group CNV comparisons between Chinese and Western breeds. Totally, 315 and 333 CNVs were detected in Chinese and Western breeds, respectively (Table 2). Among these CNVs, 186 were shared, but 129 and 147 were unique to either Chinese or Western breeds, respectively (Tables D and E in S3 File).

**Table 2:** Distribution of shared CNVs in Chinese and Western pig groups.

Number of Breed(s)	CNV numbers	
	Chinese population	Western population
≥ 1	2,278	1,547
≥ 2	964	469
≥ 3	530	133
≥ 4	315	5
≥ 5	166	—
≥ 6	89	—
≥ 7	27	—

We found that the Chinese and Western origin specific CNVRs harbor a total of 59 and 96 protein-coding genes, respectively (Tables D and E in S3 File). Gene ontology analysis showed that the CNVRs harbored genes in the Chinese breeds are mainly involved in inflammatory response and reproduction (Table F in S3 File). Selective sweep analysis also indicated that high litter size and male reproductive traits were selected in Chinese domestic breeds during domestication [33]. In contrast, an enrichment of genes involved in the regulation of muscle tissue, cell, and neuron development was only present in Western domestic pigs (Table F in S3 File).

**Fig 4:**
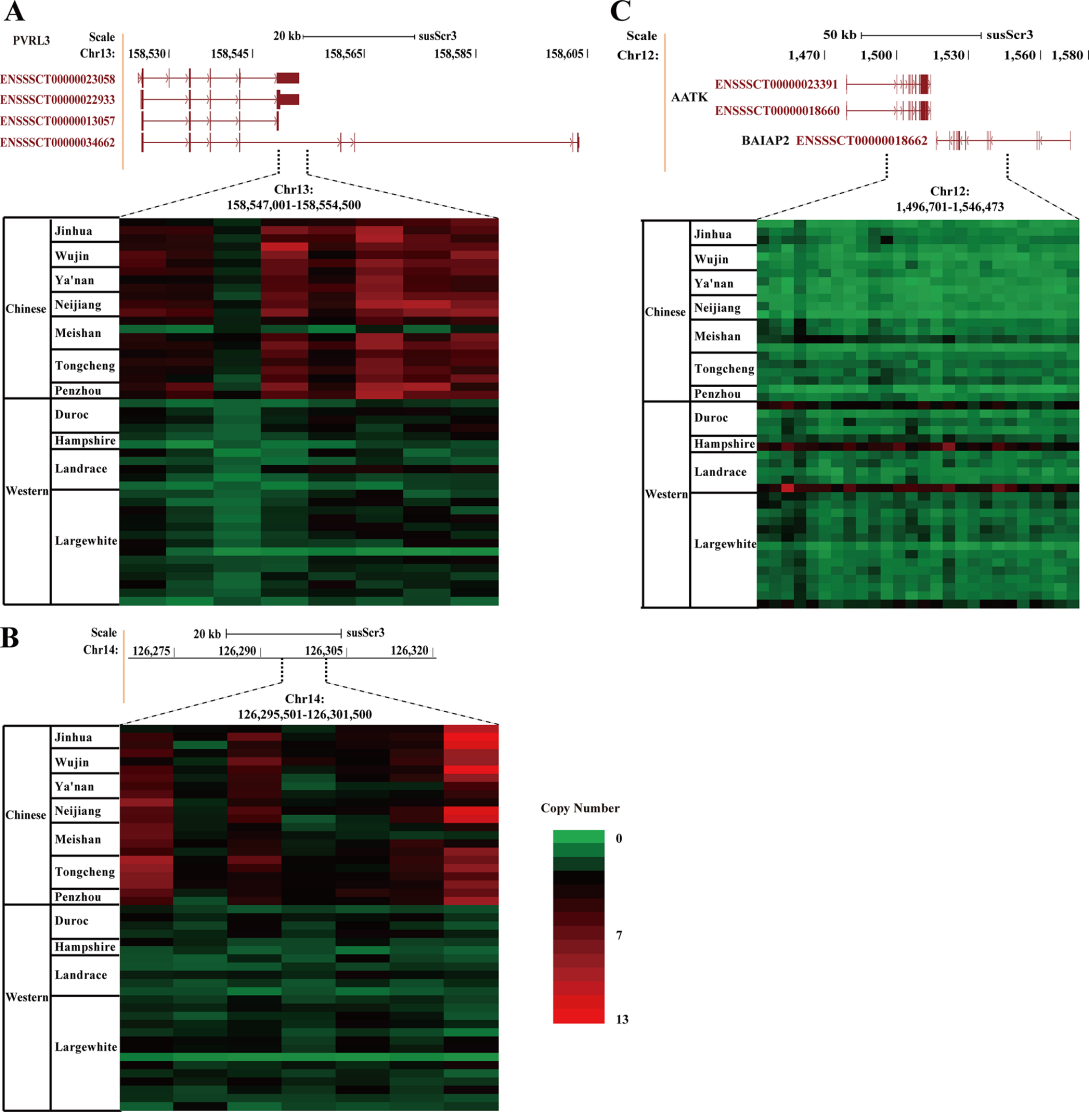
Heatmap analysis of CNV genes between Chinese and Western domestic pigs. The heatmap boxes show the sliding and nonoverlapping windows, 1 Kb in **(A)** and **(B)** and 2 Kb in **(C)**. CN values were plotted within these three regions and correspond to the different colors. On the top of the heatmap is the information of genes affected by CNVs. **(A)** A region of nearly 8 Kb (chr13: 158,547,001–158,554,500) in *PVRL3* gene had higher CN (from 5 to 9 copies), especially in Chinese domestic pigs (15 individuals / 22 totals), which was not found in Western domestic pigs. The last exon of three transcripts and the fourth intron of one transcript of this gene were affected by this CNVR. **(B)** A 6 Kb region at chromosome 14 from 126,295,501 to 126,301,500 bp had a higher CN in Chinese domestic pigs, but not in Western pigs. In addition, no genes were overlapped within this region. **(C)** The heatmap of *AATK* and *BAIAP2* genes indicated that CN gain was present in Western domestic pigs in this region, but not in Chinese domestic pigs (chr12: 1,496,701–1,546,473).

We also found that some interesting origin specific CNVRs harbored genes. One such example is the poliovirus receptor-related gene *PVRL3*, also known as *NECTIN-3*, which is located on chromosome 13 (158,524,401–158,603,720). A 8 Kb region nearby this gene had a high CN (5~9 copies) in Chinese domestic pigs (7 breeds and 15 individuals), but no CN variation was found in Western pigs (Fig 4A). This gene has 4 transcript variants and encodes the cell adhesion protein Nectin 3. We predicted that the CNVR affected different exons and introns in the four transcripts of this gene (Fig 4A). A chromosomal translocation upstream of the *PVRL3* gene significantly affects Nectin 3 expression and leads to congenital ocular defects in humans [54]. In addition, *PVRL3* as an immune gene plays an important role in the Nectin-Wave pathway and is important in cell-cell adhesion according to the gene ontology analysis. The over-expression of *PVRL3* gene could significantly inhibit tumour growth [55]. If CN gain existed in *PVRL3* gene and promoted the gene expression, Chinese domestic pigs might be more resistant to some diseases. Another CNVR was a 6 Kb region on chromosome 14 from 126,295,501 to 126,301,500 bp. This region had the highest CN gain, almost 13 copies in some Chinese domestic pigs, but no CN gain in Western domestic pigs. There was no gene annotated in this region (Fig 4B).

Additionally, the genomic region on chromosome 12 (1,496,701–1,546,473), which harbors the *BAI1*-associated protein 2 (*BAIAP2*) and apoptosis-associated tyrosine kinase (*AATK*) genes, was found to have a high CN gain in Western domestic pigs, but not in Chinese domestic pigs (Fig 4C). The most interesting function for *BAIAP2* gene, also named *IRSP53*, is that its over-expression induced filopodia formation, decreased cell adhesion and inhibited myogenic differentiation in C2C12 cells [56]. However, mutations in the conserved IMD domain of BAIAP2 abolished the inhibition of myoblast differentiation and increased the development of myotubes [56]. Moreover, many genes that promote myoblast differentiation had higher levels of expression in Landrace compared to Tongcheng pigs [57]. Thus, we predict that the CNV in the last 11 exons of *BAIAP2* gene might change the IMD domain and promote fusion of myoblasts to form multinucleate myotubes, and thus promote the rapid growth rate of muscle cells and muscle development in Western pigs compared with Chinese pig breeds. In addition to the *BAIAP2* gene, the *AATK* gene was also located in the CNVR, and except for the first exon, all the other exons of this gene had high CN gain (13 copies) only in Western domestic pigs. AATK inhibits cell proliferation, migration and also promotes apoptosis in melanoma cells [58].

The third interesting CNV-associated gene in 22 individuals was found in the exons of catenin alpha 1 gene (*CTNNA1*). The protein encoded by this gene is associated with cadherin and is a myogenesis inhibitor. A previous study showed that *CTNNA1* was expressed at higher levels in Lantang pigs, a China indigenous obese pig breed, than in Landrace pigs [59]. Thus, we predict that the CN loss in the last two exons of *CTNNA1* results in a loss of 141 amino acids and promotes pig muscle development by affecting gene expression in Western domestic pigs.

Fertilization associated genes, *WBP2* N-terminal like (*WBP2NL*) and zonadhesin (*ZAN*), were located within CNVRs identified in Chinese domestic pigs, but not in Western domestic pigs. WBP2NL promotes meiotic resumption and pronucleus formation and is involved in fertilization in humans, mice and bulls [60]. Tardif *et al*. concluded that ZAN is important for sperm-zona pellucida adhesion, which is one of the essential steps of fertilization [61]. Gene ontology analysis of CNV-associated genes revealed an enrichment of fertilization in Chinese pigs compared to Western pigs, which was consistent with the higher litter size observed in Chinese domestic pigs (Table F in S3 File).

The high CNV differences between Chinese and Western breeds can be attributed to three potential reasons. Firstly, the history of domestication from wild boar to Chinese or Western domestic pigs was different. European and Asian wild boars were derived from the wild pigs originating in Southeast Asia and the phylogenetic split occurred between them during the mid-Pleistocene epoch 1.6–0.8 Myr ago [34]. Subsequently, domestication occurred independently in Western and Chinese breeds with different climates, geographical positions, and human hunting, which contributed to great diversities between them [33, 53, 62]. Secondly, different traits were selected in Chinese and Western breeds. With the long term artificial selection for reproduction, growth and disease resistance, the beneficial CNVs were conserved during domestication. For example, CNV-associated muscle development and growth rate for Western breeds might be preserved during domestication and long-term selection, and CNVs involved in resistance and tolerance of some diseases were maintained in Chinese breeds. Thirdly, the selection of Western breeds for commercialization gradually led to purification and decreased individual variation [12]. Conversely, a large population size and diverse origin occurred in Chinese pigs [34], which might have led to higher CNV counts and length in Chinese breeds than in Western breeds according to our results. Accordingly, we speculated that the CNVs were variable in Chinese and Western domestic pigs because of independent domestication, resulting in some different traits among them. The unique CNVs found in our results might contribute to disease resistance of Chinese domestic pigs and fast growth of Western domestic pigs, and facilitate our understanding of the trait differences between Chinese and Western domestic pigs during domestication.

## Conclusions

In this study, we used next generation sequencing data to detect CNVRs in the porcine genome using 49 pigs, representing a large population for CNVRs scanning. CNVseq, CNVnator and other strict standards were used to enhance the credibility of the results. A large number of novel CNVRs and associated genes involved in immunity, olfaction and growth for pigs were identified. Different CNVs between Chinese and Western breeds were analyzed, and 129 and 147 CNVs were found to exist only in Chinese and Western breeds, respectively. Gene function analysis in the CNVRs revealed that an enrichment of inflammatory response and fertilization exist only in Chinese domestic pigs, and an enrichment of muscle tissue, cell, and neuron is specific to Western domestic pigs. We also found some CNV-associated genes were involved in immunity, fertilization and muscle development, such as *PVRL3*, *AATK*, *BAIAP2*, *WBP2NL*, *ZAN* and *CTNNA1* genes, which might lead to the observed phenotypic differences between Chinese and Western breeds. The data from the extensive analysis of CNVs provide some genetic markers and valuable insights for further research of Chinese and Western pig breeds during domestication.

## Supporting Information

S1 File Supporting tables. Table A, Information of samples used in our analysis. Table B, Information of identified CNVRs in 47 individuals. Table C, Densities of CNVRs in each autosome.
(XLSX)

S2 File Supporting tables. Table A, Information of the primers used in qPCR analysis of the 28 CNVRs chosen to for validation. Table B, Exons and introns of genes affected by the identified CNVRs. Table C, Gene Ontology analysis of genes in identified CNVRs. Table D, CNV associated genes involved Olfactory. Table E, CNV associated genes involved Immune.
(XLSX)

S3 File Supporting tables. Table A, CNVRs found in Chinese domestic pigs. Table B, CNVRs found in Western domestic pigs. Table C, Comparison of identified CNVRs between Chinese and Western domestic pigs. Table D, CNVRs and associated genes unique to Chinese domestic pigs. Table E, CNVRs and associated genes unique to Western domestic pigs. Table F, Gene ontology analysis of CNV-associated genes in Chinese and Western breeds, respectively.
(XLSX)

S4 File Supporting Figure. CNVRs near *KIT* gene locus. The *KIT* gene region associated with high CN leads to the dominant white color in Western pigs. **(A)** The Log2 Ratio CNV graph near the *KIT* gene region in Hampshire and LargeWhite pigs was generated by the ggplot package. Red plots indicate the value of log2 (reads count of test/reads count of reference) in each window. CN gain was found in a large CNV region of 43.2–43.8 Mb in LargeWhite pigs and two CNV regions of 43.3–43.4 Mb and 43.6–43.8 Mb in Hampshire pigs. **(B)** CN values were estimated using the qPCR method in two regions near the *KIT* gene. The different cycles indicated the CN values in different individuals from three Western breeds. LargeWhite and Landrace individuals showed CN gain ranging in size from 3 to 11 in the *KIT*_1 region and 6 to 21 in the *KIT*_2 region, but no CNV was found in Duroc individuals.
(TIF)

S5 File Supporting Figure. CN values predicted and observed near *ATL2* gene locus. The left picture indicated that there was no CN gain in this locus, which did not agree with the predicted CN in our result. However, the predicted and observed CN was similar in Tongcheng four pigs in right picture.
(TIF)
