# Supplementary figures and images for: Genome Wide Distributions and Functional Characterization of Copy Number Variations between Chinese and Western Pigs
Source: PLoS One. 2015 Jul 8;10(7):e0131522. doi: 10.1371/journal.pone.0131522 (PMC4496047; doi:10.1371/journal.pone.0131522)

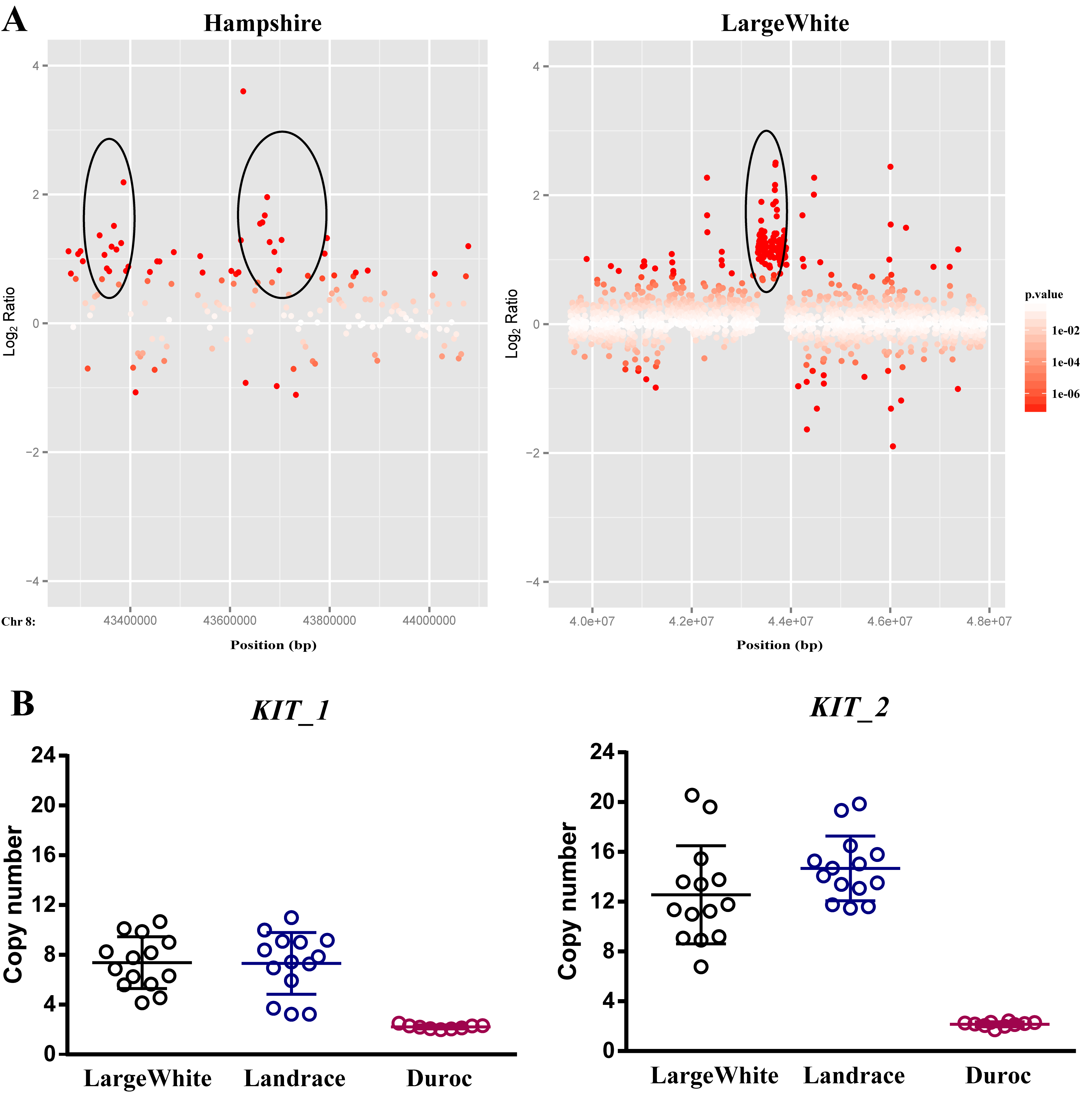

Supplement: S4 File — CNVRs near KIT gene locus. The KIT gene region associated with high CN leads to the dominant white color in Western pigs. (A) The Log2 Ratio CNV graph near the KIT gene region in Hampshire and LargeWhite pigs was generated by the ggplot package. Red plots indicate the value of log2 (reads count of test/reads count of reference) in each window. CN gain was found in a large CNV region of 43.2–43.8 Mb in LargeWhite pigs and two CNV regions of 43.3–43.4 Mb and 43.6–43.8 Mb in Hampshire pigs. (B) CN values were estimated using the qPCR method in two regions near the KIT gene. The different cycles indicated the CN values in different individuals from three Western breeds. LargeWhite and Landrace individuals showed CN gain ranging in size from 3 to 11 in the KIT_1 region and 6 to 21 in the KIT_2 region, but no CNV was found in Duroc individuals. (TIF) [file pone.0131522.s004.tif]

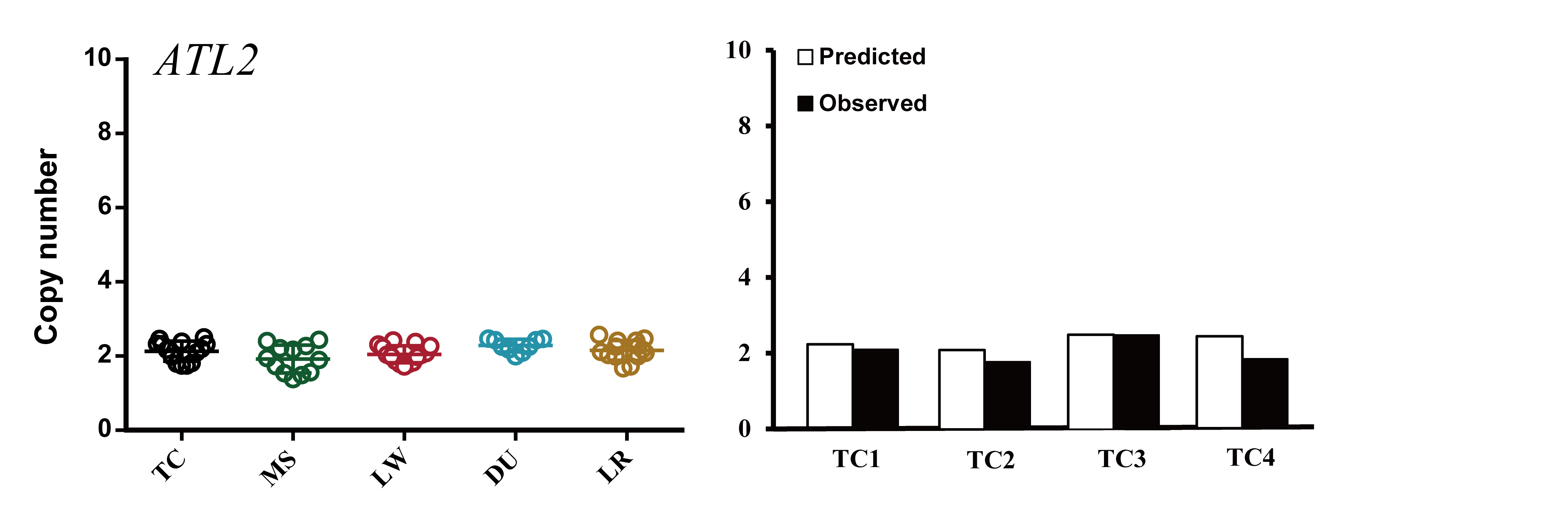

Supplement: S5 File — CN values predicted and observed near ATL2 gene locus. The left picture indicated that there was no CN gain in this locus, which did not agree with the predicted CN in our result. However, the predicted and observed CN was similar in Tongcheng four pigs in right picture. (TIF) [file pone.0131522.s005.tif]
